# Health-related quality of life of patients following selected types of lumbar spinal surgery: A pilot study

**DOI:** 10.1186/1477-7525-5-71

**Published:** 2007-12-28

**Authors:** Karen L Saban, Sue M Penckofer, Ida Androwich, Fred B Bryant

**Affiliations:** 1 Niehoff School of Nursing, Loyola University Chicago, Chicago, IL, USA; 2 Department of Psychology, Loyola University Chicago, Chicago, IL, USA

## Abstract

**Background:**

Over 500,000 spinal surgeries are performed annually in the United States. Although pain relief and improved health-related quality of life (HRQOL) are expectations following lumbar spinal surgery, there is limited research regarding this experience from the individual's perspective. In addition, no studies have examined the HRQOL of persons who have had this surgery using a comprehensive approach. The intent of this study was to address this deficiency by an assessment of both the individual and environmental factors that impact perceived HRQOL using the Wilson and Cleary Model for Health-Related Quality of Life in persons who have undergone lumbar spinal surgery.

**Methods:**

This was a pilot study of 57 adult patients undergoing elective lumbar spinal surgery for either herniated disk and/or degenerative changes. Individuals completed questionnaires measuring perceived pain, mood, functional status, general health perceptions, social support and HRQOL preoperatively and three months following surgery. Descriptive statistics, dependent t-tests, and MANOVAs were used to describe and compare the differences of the study variables over time.

**Results:**

Preliminary results indicate overall perceived physical HRQOL was significantly improved postoperatively (t [56] = 6.45, p < .01), however, it was lower than the published norms for patients with low back pain. Both functional disability (t [56] = 10.47, p < .001) and pain (t [56] = 10.99, p < .001) were significantly improved after surgery. Although levels of fatigue and vigor were also significantly improved after surgery, both were less than the published norms. There was no change in the level of social support over time; however, level of support was consistent with that reported by patients with chronic illness.

**Conclusion:**

Although perceived physical HRQOL was significantly improved three months postoperatively, fatigue and lack of vigor were issues for subjects postoperatively. Excessive fatigue and low vigor may have implications for successful rehabilitation and return to work for patients following lumbar spinal surgery. Further research is needed with a larger sample size and subgroup analyses to confirm these results.

## Background

Lumbar spinal surgery is one of the most common types of surgeries performed in the United States with over 500,000 surgeries performed for lumbar herniated disks and lumbar spinal stenosis in 2004 [1]. Numerous studies have reported the clinical outcomes of spinal surgery. However, many studies have defined success rates in terms of medically-related outcomes, such as fusion rates and radiographic evidence, rather than the patient's perspective. Studies have demonstrated that patients' perspectives of their clinical outcomes are not necessarily the same as those of their clinicians'[2]. Although pain relief and improved health-related quality of life (HRQOL) are patient expectations following lumbar spinal surgery, there is limited research regarding this experience from the individual's perspective. In addition, no studies have examined the HRQOL of persons who have had this surgery using a comprehensive approach. The intent of this study was to address this deficiency by an assessment of both the individual and environmental factors that impact perceived HRQOL, using the Wilson and Cleary Model for Health-Related Quality of Life, in persons who have undergone lumbar spinal surgery.

**Figure 1:**
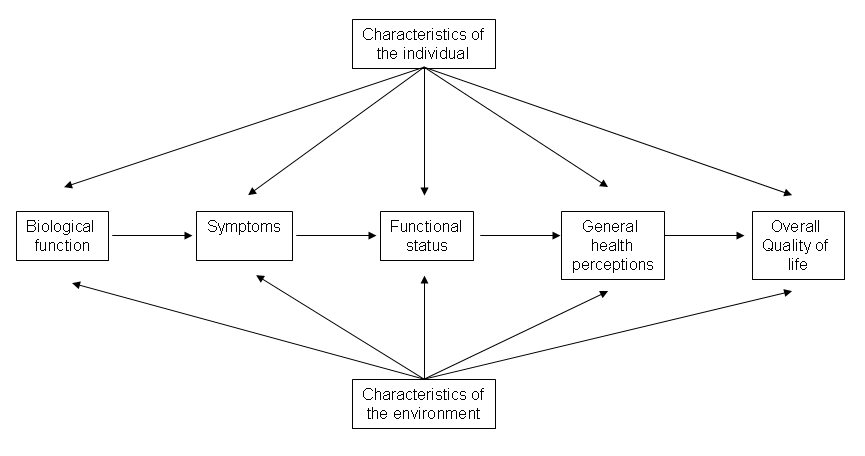
**Revised Wilson and Cleary Model for Health-Related Quality Life**. Revised Wilson and Cleary Model for Health-Related Quality of Life. Ferrans, C. E., Zerwic, J. J., Wilbur, J. E., & Larson, J. L. (2005). Conceptual model of health-related quality of life. *Journal of Nursing Scholarship, 37*, 336–342. Adapted from Wilson, I.B., & Cleary, P.D (1995). Linking Clinical Variables with Health-Related Quality of Life: A Conceptual Model of Patient Outcomes. *JAMA. 273*, 59–65. Copyright *JAMA*. Used with permissions.

Using a framework in quality of life research is important because it promotes the selection of appropriate measurement variables and identifies potential links between variables within the complex construct of quality of life. Wilson and Cleary published their conceptual model of quality of life in JAMA in 1995 [3] and it was later revised by Ferrans et al [4] (Figure 1). This model was developed in order to help explain the relationships of clinical variables that relate to quality of life. The authors of the model present it as taxonomy of patient outcomes that link the characteristics of the individual to the characteristics of the environment. The model proposes causal linkages between five different types of patient outcome measurements. The first variable, the biological and physiological variable is considered the most basic. It includes such measurements as laboratory tests, blood pressure and physical examination. The second variable is symptom status. It consists of physical, emotional and psychological symptoms that the patient may subjectively experience. The third variable in the model is functional status which refers to the patient's ability to perform certain tasks or functions. Functional status is usually subjectively reported by the patient but can also be assessed by others. The fourth variable, general health perceptions is the global perception of the individual of his general health state and takes into account the weights and values that the patient attaches to symptoms or functional abilities. Finally, QOL is the patient's overall satisfaction with life. The arrows represent dominant causal relationships. Reciprocal relationships between the variables are recognized to exist but are not represented. Since the revised Wilson and Cleary model incorporates individual characteristics with environmental characteristics, it is a useful model for guiding QOL research, especially in patients with lumbar spinal disease since their recovery may be affected by both internal factors (such as physiological variables, personality, values and preferences) as well as characteristics of the environment (such as social support).

### Characteristics of individual

Several socio-demographic variables are associated with the incidence as well as treatment outcome of patients with low back pain. For instance, patients with higher levels of education tend to have a decreased risk of developing low back pain [5]. This finding may be related to type of work of patients with higher levels of education having less physically labor intensive jobs. Some studies have correlated demographic information with clinical outcomes of lumbar spinal surgery. For instance, one study reported that subjects who reported the best improvements in physical functioning and ability to walk after surgery were male and younger [6]. Race was uncommonly reported in the spinal surgery literature. However, in the published studies where race was reported, racial disparity existed with most samples being predominately white [7].

### Characteristics of environment

Degree of social support is considered to represent an aspect of the environment in the revised Wilson and Cleary model of HRQOL [4]. Although several studies have considered the role of social support in recovery and HRQOL outcomes [8], only one study was found that examined social support as a predictor of the surgical outcome of patients undergoing spinal surgery [9]. This study, although it did not specifically measure quality of life, found that subjects who had severe psychological strain and lack of social support had poor surgical outcomes.

### Biological function

Some studies have demonstrated that subjects with chronic low back pain tend to be sicker than the general population with a higher incidence of associated comorbidities such as depression, anxiety, sleep disturbances, and headaches [10,11]. Hestbaek et al [11], suggested that, based on a literature review of comorbidities and low back pain, that low back pain may be part of a disease cluster in some individuals. Other studies have not found a higher incidence of comorbidities in back sufferers [5]. In addition, obesity has been associated with poorer outcomes of spinal surgery [12] due to difficulties in mobilizing after surgery as well as impaired wound healing.

### Symptoms: pain and mood

The primary complaints of patients undergoing lumbar spinal surgery are back pain and radicular pain accompanied by leg weakness. The goal of spinal surgery is to either completely alleviate pain or to greatly minimize it. Numerous studies have reported measures of level of pain before and after lumbar spinal surgery [13]. Postoperative reports of pain in the literature varied depending upon such factors as the type and extent of surgery, comorbidities, and time since surgery. However, most studies reported improvement in pain postoperatively. For example, in one prospective study of 281 patients who underwent lumbar surgery for degenerative changes, herniated disks, instability, or spinal stenosis, 80% reported that their pain intensity level had improved at least moderately one year after surgery [14].

The literature related to other symptoms (such as depression and anxiety) in patients undergoing lumbar spinal surgery was much less robust than the pain literature. Although there is literature to suggest that back pain is often associated with mood disorders [15], no studies were found that considered whether or not mood improved after lumbar spinal surgery.

### Functional status

Wilson and Cleary [3] defined functional status as the ability of the patient to perform certain tasks and functions. The functional status variable includes physical functioning, social functioning, emotional functioning and role functioning. Functional status has been measured as both a predictor variable and outcome measurement in the spinal surgery population. Many studies have reported improvement in functional status of patients undergoing lumbar spinal surgery [16]. However, functional status as an outcome variable is often measured in terms of the ability to return to work [7,17]. Most of these studies found return to work status to be highly variable and dependent upon such factors as preoperative disability level, age, type of work and type and extent of surgical procedure.

### General health perceptions

According to Wilson and Cleary [3], general health perceptions take into account satisfaction with health as well as how symptoms and functional abilities are valued. No studies were found that specifically measured health perceptions in patients undergoing lumbar spinal surgery.

### Health-related quality of life

A number of studies were conducted measuring the HRQOL in patients undergoing spinal surgery [18]. The most common measures of HRQOL in these studies were the SF-36 [19], the Roland Morris Disability Questionnaire [20], and the Stauffer-Coventry Index [21]. Overall, research indicated that patients undergoing lumbar spinal surgery did demonstrate improvements in HRQOL postoperatively. However, there was wide variance in how HRQOL was conceptualized and measured. In addition, no studies were found that evaluated HRQOL within a comprehensive framework. Therefore, the overall purpose of this study was to address this deficiency by examining HRQOL of patients undergoing lumbar spinal surgery guided by the revised Wilson and Cleary QOL Model [4].

## Methods

### Design

This longitudinal one-group pretest-posttest study was part of a larger study that examined the relationships between changes in social support, pain, mood, functional status, perceived health status, patient expectations, optimism, and perceived QOL of subjects undergoing lumbar spinal surgery for either herniated disk and/or degenerative changes [22,23]. This report focuses on the changes in social support, pain, mood, functional status, perceived health and perceived HRQOL after lumbar spinal surgery.

### Setting and sample

Using a consecutive convenience sample, the study was conducted at five Midwestern hospitals in the United States. Surgical technique and procedures were considered similar among these sites. The inclusion criteria were patients undergoing elective lumbar spinal surgery for the first time for degenerative changes and/or herniated disks, age 18 years or older, with the ability to read and write English. Patients undergoing lumbar spinal surgery for degenerative changes and/or herniated disks were chosen because they were expected to make at least some symptom and functional improvements by 3 months postoperatively. Patients with cancer, spinal cord injury, cauda equina syndrome, and more than two levels of fusion were excluded from the study in order to control for significantly different recovery trajectories. Both genders as well as different ethnic groups were included in the study based upon meeting the inclusion criteria.

Ninety-four patients were approached for participation in the study. Of these, 73 (77.6%) completed the preoperative questionnaire. Postoperatively, 57 (78%) subjects completed the follow up questionnaire resulting in a total sample size of 57 for analysis.

### Procedure

The study was approved by the university and hospital institutional review boards. A one-page information sheet inviting patients who met the inclusion criteria to participate in the study was made available in waiting rooms and exam rooms. In addition, potential subjects were identified by the surgeons and clinic nurses.

Potential subjects were informed of the purpose, risk/benefits of the study, and were invited to participate in the study. After obtaining informed consent, subjects completed a preoperative questionnaire booklet 2–14 days prior to surgery and then a postoperative questionnaire booklet approximately 3 months after surgery.

### Variables and instruments

The revised Wilson and Cleary Model for Health-Related Quality of Life [4] provided the basis for the selection of the variables studied. The variables and their corresponding measurements tools are summarized in Table 1.

**Table 1:** Health-Related Quality of Life Variables

Revised Wilson and Cleary HRQOL Concepts	Study Variable	Measurement Tool
Characteristics of individual	Age, gender, marital status	Demographic questionnaire
Characteristics of environment	Social support	Medical Outcomes Study – Social Support
Biological function	Type of surgery, number of spinal levels, BMI	Medical Chart Review, Medical History Form
Symptoms	Mood	Profile of Moods State (POMS-Brief)
	Pain	Numeric Pain Rating Scale
Functional status	Disability level	Oswestry Disability Index for Low Back Pain
General health perceptions	Overall health	Overall health item
Quality of life	Perceived physical HRQOL	SF-12 Physical component summary
	Perceived mental HRQOL	SF-12 Mental health component summary

### Characteristics of the individual

The investigator developed a demographic form to collect subject demographic information such as patient age, gender, marital status, race, work status, and educational level.

### Characteristics of the environment

The Medical Outcomes Study (MOS) Social Support Survey [24] was used to measure perceived availability of social support. The MOS is a structured, self-report questionnaire with responses to each item given on a 5-point Likert scale from 1 = none of the time to 5 = all of the times. The total score generated from nineteen items was used in the analysis for this study. In the initial study using the MOS in 2987 subjects, the total Cronbach's alpha was 0.97 [24]. The MOS demonstrated excellent test-retest reliability (0.78) taken at a one-year interval and high convergent and discriminant validity [24]. No studies were found that used the MOS in the spinal surgery population, however the tool seemed be appropriate for this group of subjects. For this study, Cronbach's alpha coefficients for the total scores for the MOS were good (preoperatively 0.95 and postoperatively 0.96).

### Biological function

A medical chart review form was developed by the investigator to collect biological and physiological variables pertinent to this study including comorbidities, presence of obesity as measured by body mass index over 30, and type of surgery.

### Symptom status: pain and mood

The Numeric Pain Rating Scale (NPRS) was used to assess degree of back pain. The NPRS is a 0 to 10 point scale in which 0 is considered no pain and 10 is the worst pain possible. The instrument has been used extensively in a wide variety of settings and has been validated with low back pain patients [25]. Level of pain was measured both preoperatively and postoperatively.

The Profile of Mood States-Brief Form (POMS-Brief) [26] was used to assess affective mood states. The POMS-Brief, developed from the longer 65-adjective POMS, is a commonly used measure of psychological distress and has been found to be particularly useful in measuring changes in mood over time and therefore was appropriate in this longitudinal study. The 30-adjective POMS-Brief examines the same six mood states of the longer POMS: Tension-Anxiety, Depression-Dejection, Anger-Hostility, Vigor-Activity, Fatigue-Inertia and Confusion-Bewilderment. Scores for each of the six subscales range from 0–20 with higher scores indicating higher distress except for the subscale of Vigor-Activity which is negatively scored. A total mood score is obtained by adding the scale scores of Tension-Anxiety, Depression-Dejection, Anger-Hostility, Fatigue-Inertia, and Confusion-Bewilderment and subtracting the scale score of Vigor-Activity. The total mood score ranges from 0–80 (from least disturbed to most disturbed). According to the POMS Manual, internal consistency estimates for the POMS were found to be satisfactory nearing .90 or above [26]. Test-retest reliability coefficients were reported to range from .61 to .69 [27]. For this study, reliability for the total mood disturbance scores were good (preoperative Cronbach's alpha = 0.90 and postoperative Cronbach's alpha = 0.92) and were consistent with those reported in previous studies [26,28].

### Functional status

Disease-specific functional status was measured using the Oswestry Disability Index for Low Back Pain (ODI) Version 2.0 [29]. The ODI is a self-administered tool that consists of 10 items, each with six possible choices ranging from normal functioning to inability to function. The ODI measures the patient's ability to function in areas of daily living that are most likely impaired by patients suffering from low back pain such as ability to walk and lift objects. The total score provides a disability score: 1) 0–20 = Minimal disability; 2) 20–40 = Moderate disability; 3) 40–60 = Severe disability; 4) 60–80 = Crippled and 5) 80–100 = Bed-bound or exaggerating symptoms.

Test-retest scores with an interval of 4 days was found to be high (r = 0.91) [30]. Internal consistency using Cronbach's alpha was shown to be acceptable ranging from 0.71 to 0.87 in a number of studies i.e. [30]. For this study, Cronbach's alpha was good (0.78 preoperatively and 0.80 postoperatively).

### General health perceptions

General health perceptions were evaluated at each time point with a single item that asked the respondent to rate their overall health as "excellent", "very good", "fair" or "poor". Studies have supported the reliability and validity of using a single-item indicator to measure such variables as well-being and health perceptions [31].

### Health-related quality of life

Health-related quality of life was measured with the SF12v2 [32]. The SF12v2 is a generic measure that consists of 12 items and provides scores for eight health concepts as well as two summary outcomes for physical health and mental health. The SF12v2 was derived from the SF-36, one of the most widely used health surveys in the world [32].

Published reliability coefficients range from 0.73 to 0.87 across all eight subscales of the SF-12v2 [32]. For the two summary scales, PCS-12 and MCS-12, reliability estimates were 0.89 and 0.86 respectively. No studies were found that used the SF-12 or SF-12v2 in the spinal surgery population; however, many studies have used the SF-36.

The authors of the S12v2 recommend intraclass correlations for estimating test-retest reliability for the SF-12 PCS and MCS [32]. For this study, test-retest reliability based on intraclass correlations between preoperative and postoperative measurements were PCS = 0.44 and MCS = 0.47. These reliabilities may be low due to a change in the patients' health after surgery as well as a three-month period between test administrations.

### Data analysis

Data was entered into the statistical analysis program, SPSS 14.0 (SPSS Inc., Chicago, IL) for each instrument. Missing data per subject ranged from 0.5% to 11.3% with a mean of 2.9% (N = 57). Upon examination of each question, no patterns of missing data were noted. Missing data in key variables were replaced with values using a multiple imputation procedure based upon a regression model.

Descriptive statistics, dependent t-tests, and analysis of variance (ANOVA) were used to describe and compare differences between the preoperative and postoperative variables. Multivariate analysis of variance (MANOVA) was used to detect changes over time in subscales.

## Results

### Characteristics of individual

Subjects (N = 57) averaged 53.4 years of age with age ranging from 21 to 84 years old. For patients undergoing surgery for primarily herniated disk(s), (N = 34, 60%), the mean age was lower (M = 48.81, SD = 12.93). As expected, the mean age for patients undergoing surgery for spinal stenosis and degenerative changes (N = 10, 17%) was higher (M = 65.7, SD = 6.61). Patients undergoing lumbar fusion (N = 13, 23%) had a mean age of 56.53 (SD = 11.30). There were slightly more women (N = 30, 52.6%) than men (N = 27, 47.4%) who participated in the study. Most subjects were married (N = 40, 70.2%). The majority were white (N = 51, 89.5%) and had at least some college education. Only 19.3% (N = 11) of participants were working full-time without any restrictions prior to surgery. Preoperatively, 36.8% (N = 21) of subjects indicated that they had decreased their work hours or were not able to work because of their back problem.

### Characteristics of environment

The variable of social support represented an aspect of the characteristic of the environment in the model. Overall, subjects reported moderate levels of social support both preoperatively (M = 68.21, SD = 20.91) and postoperatively (M = 67.53, SD = 22.90) (Table 2). A paired t-test revealed no significant difference between the preoperative and postoperative MOS total scores (t [54] = .132, p = .895).

**Table 2:** Preoperative and Postoperative Results of Quality of Life Variables (N = 57)

	Preoperative	Postoperative	p-Value
Social Support (M, SD)	68.21 ± 20.91	67.53 ± 22.90	.895
			
Pain (M, SD)	7.00 ± 1.80	3.19 ± 2.30	**<.001**
			
Mood (M, SD)			
Total Mood	28.57 ± 19.84	16.35 ± 11.98	**<.05**
Tension	7.36 ± 4.36	4.13 ± 4.12	**<.001**
Depression	5.18 ± 4.80	4.26 ± 4.85	.469
Anger	5.15 ± 4.60	3.91 ± 4.57	.175
Vigor	4.05 ± 3.75	7.42 ± 4.18	**<.001**
Fatigue	9.43 ± 5.32	7.12 ± 4.86	**<.05**
Confusion	5.13 ± 3.11	4.35 ± 3.14	.469
			
Functional status (M, SD)	51.31 ± 15.48	23.89 ± 15.96	**<.001**
			
Overall Health (M, SD)	3.33 ± 1.15	3.36 ± 1.17	.898
			
HRQOL (M, SD)			
Physical Component	29.39 ± 8.10	38.66 ± 11.99	**<.001**
Mental Component	46.43 ± 11.90	49.99 ± 11.29	.120
Physical Functioning	29.43 ± 9.20	37.62 ± 12.19	**<.001**
Role Physical	30.85 ± 8.22	38.66 ± 11.04	**<.001**
Bodily Pain	29.05 ± 9.06	41.68 ± 11.05	**<.001**
General Health	44.01 ± 12.45	46.40 ± 13.99	.136
Vitality	41.16 ± 9.92	44.69 ± 14.69	**<.05**
Social Functioning	37.09 ± 12.31	45.69 ± 12.14	**<.001**
Role Emotional	41.76 ± 12.37	44.41 ± 12.11	.131
Mental Health	42.60 ± 11.90	47.67 ± 11.62	**<.05**

* Bolded text is significant

### Biological function

Subjects reported a wide variety of comorbidities including hypertension (33.3%, N = 19), osteoarthritis (21%, N = 12), and diabetes (10.5%, N = 6) being the most common. Most subjects were either overweight or obese (70.2%, N = 40), which is a common risk factor in the development of back pain. The type of surgical procedures performed included lumbar microdiscectomy (N = 34, 59.7%), lumbar fusion (N = 13, 22.8%) and lumbar laminectomy (N = 10, 17.5%). Most participants (N = 41, 71.9%) had only one spinal segment operated on and the majority (N = 46, 80.7%) did not require instrumentation.

### Symptom status: pain and mood

The variables of pain and mood corresponded to symptom status in this study. The Numeric Pain Rating Scale (NPRS) was used to measure degree of pain on a scale of 0 to 10 with 0 being no pain and 10 being extreme pain. Pain decreased from a mean of 7.0 preoperatively to a mean of 3.19 postoperatively (Table 2). The decrease in pain from the preoperative to the postoperative period was significant (t [56] = 10.99, p < .001).

The POMS-Brief Form was administered both preoperatively and postoperatively to measure mood. The overall total mood disturbance score was calculated as well as six subscales measuring tension-anxiety, anger-hostility, vigor-activity, fatigue-inertia, confusion-bewilderment, and depression-dejection (Table 2). A paired t-test revealed that there was a significant improvement between preoperative total mood and postoperative total mood (t [56] = -4.009, p < .001). A repeated measures general linear model was used to test for differences between time periods on the subscales. Results indicated an overall difference within subjects for time (F [6,45] = 12.257, p < .001. Univariate analysis revealed that the subscale scores of tension (F [1, 50] = 21.345, p < .01), vigor (F [1, 50] = 25.714, p < .01), and fatigue (F [1, 50] = 4.613, p < .05) significantly improved from the preoperative to postoperative time periods. There were no significant differences between time periods for anger, confusion, and depression (Table 2).

### Functional status

Preoperatively, the ODI mean was 51.3 indicating severe disability in functional status (Table 2). Postoperatively, the ODI mean improved significantly (t [56] = 10.472, p < .001) to 23.89 indicating moderate disability in functional status.

### General health perceptions

General health perception did not change significantly between the preoperative and postoperative periods (Table 2) (t [56] = -.129, p = .898).

### Health-related quality of life

Health-related quality of life was measured with the SF-12v2 that elicited a summary score for Physical Component Summary (PCS-12) and Mental Component Summary (MCS-12). In addition, eight subscale scores (physical functioning, role physical, bodily pain, general health, vitality, social functioning, role emotional and mental health) were calculated for both the preoperatively and postoperative data. Means for the summary scores as well as all eight subscales improved from the preoperative to postoperative period (Table 2). A paired t-test demonstrated a significant improvement in the PCS (t [56] = -6.454, p < .001). However, no significant differences were found between the preoperative and postoperative MCS (t [56] = -1.519, p = .120). A repeated measures general linear model was used to calculate differences between time periods on each of the eight subscales. Results indicated that there was an overall significant difference within subjects between preoperative and postoperative time periods (F [8, 49] = 7.677, p < .001). Univariate analysis revealed significant differences between time periods for the physical functioning (F [1,56] = 27.917, p < .01), role physical (F [1,56] = 28.283, p < .01), bodily pain (F [1,56] = 64.150, p < .01), social functioning (F [1,56] = 22.318, p < .01), and mental health (F [1,56] = 10.769, p < .01). The subscales of general health, vitality and role emotional did not show significant differences between the time periods.

## Discussion

A strength of this study is that it is the first study to examine the HRQOL in persons following lumbar spinal surgery using a theoretical framework. According to the revised Wilson and Cleary's Model for Health-Related Quality of Life [4], the biomedical paradigm, which focuses on disease and pathology, is linked to the social science paradigm that encompasses dimensions of functional abilities and overall well-being. In this study, spinal disease not only affected the subject's physiological processes, but also influenced the social paradigm of both role and functional abilities.

Characteristics of the sample (individual and biologic function) were similar to other studies of patients undergoing lumbar spinal surgery in terms of reported prevalence of obesity [33] and most commonly associated comorbidities [34]. The demographics of the study sample were also similar to other studies of spinal surgery patients in relation to age, marital status, educational status, race [7], and gender [18]. The wide age range (21 to 84 years) of subjects in this study may have had implications for HRQOL results. Age was somewhat positively correlated (r = .351, p < .01) with the Mental Health Component (MCS) of the SF-12v2 but was not significantly correlated with the Physical Component Summary (PCS) score (r = .157, p > .05). In other words, older subjects tended to report higher levels of mental health HRQOL but age did not seem to make a difference in terms of physical HRQOL. The finding that age is positively correlated with higher levels of mental HRQOL is consistent with the literature of older persons in the general population reporting higher levels of overall HRQOL and well-being than younger persons [35]. Other studies have reported that older age is negatively correlated with physical HRQOL of patients undergoing lumbar spinal surgery [6,7,36,37]. It may be that these differences were not able to be detected because of our small sample size. More research is needed to further examine the role of age as it related to spinal surgery type and perceived HRQOL.

Race was uncommonly reported in the spinal surgery literature. However, in the published studies where race was reported, racial disparity existed with most samples being predominately white [7]. The sample in this study consisted of 89.5% whites, 8.9% blacks and 1.8% American Indian. This racial disproportion may be related to healthcare access, resource allocation or the regional demographics of where the study was conducted. Further research that considers race is needed in future studies of spinal surgery outcomes. Of the published studies found that reported on patients undergoing various lumbar spinal procedures, consistent with this study, the majority of subjects underwent lumbar microdiscectomies [14]. A sample consisting of subjects undergoing only one type of lumbar surgery (i.e. lumbar microdiscectomy) would have been ideal. In addition, it was suggested in one study that compared quality of life between subjects undergoing surgery for decompression for spinal surgery with spinal instrumentation (N = 15) to those without spinal instrumentation (N = 8) that the use of spinal instrumentation may positively influence postoperative HRQOL [38] by allowing for better fusion rates. It may be important in future studies to conduct subgroup analysis considering various types of surgeries and use of spinal instrumentation using sample sizes that are adequately powered.

In terms of environment, to the authors' best knowledge, this study is the first to use the Medical Outcomes Study (MOS) Social Support Survey to measure level of social support in patients undergoing lumbar spinal surgery. Only one study was found that examined social support as a predictor of surgical outcome of lumbar discectomy and found that subjects who had severe psychological strain and lack of social support had poor surgical outcomes [9]. Level of social support has also been found to positively influence compliance with prescribed medical regimens [39] as well as overall quality of life in patients undergoing surgery [40]. In contrast, social support has also been found to negatively influence outcomes after lumbar spinal surgery if the family member excessively reinforces the pain [41] or the patient overly relies on available support [42]. A comparison of mean scores for the MOS suggests that lumbar spinal surgery patients in this study reported moderately high levels of social support similar to patients with chronic conditions (M = 70.1) [24]. Interestingly, level of social support did not change after surgery in contrast to a study of patients who underwent cardiac surgery who exhibited significantly decreased levels of social support three months after surgery [8]. The finding in this study may be attributed to possible differences in the levels of associated comorbidities and/or surgical recuperation of these two populations. On the other hand, social support levels remaining high after back surgery may be an indication of an over reliance on social support systems. Additional research considering the family or caregiver's perspective may be important to consider in future studies.

Although functional status was significantly improved after surgery, subjects remained moderately disabled three months after surgery. This finding is consistent with the literature regarding spinal surgery [43]. Yukawa et al [43] reported that in a study of 62 subjects who underwent a laminectomy for spinal stenosis, functional status was significantly improved 6 to 18 months postoperatively. Similar improvements in functional status were reported in a study of patients following anterior lumbar fusion [44]. Further research is needed to clarify the expected time of optimal functional recovery following different types of spinal surgery.

In terms of symptom status, subjects experienced significant improvement in pain relief following surgery. This is also supported by other studies of spine surgery patients that examined pain relief [45]. Jang & Lee [45] reported that in a sample of subjects undergoing minimally invasive lumbar fusion level of pain as measured on the NPRS significantly improved from a score of 7.5 to 2.3 postoperatively. However, no studies were found that considered the quality, duration and frequency of pain as well as use of pain relieving medications in the months following spine surgery. A more comprehensive measurement of pain may be important in future studies of patients undergoing spinal surgery.

Despite overall mood being significantly improved after surgery, subjects still reported lower levels of vigor and higher levels of fatigue as compared to published norms [26]. This may have been a result of continued recovery from surgery at 3 months postoperative or it could have been related to subjects feeling greater fatigue as they returned to normal activities. Some studies have found that chronic low back pain is associated with higher rates of mood disturbances [15]. No studies were found that considered whether or not mood improved after lumbar spinal surgery.

In the current study, the postoperative total mood disturbance score was slightly higher than a sample of patients who had undergone coronary artery bypass surgery one month previously [46] and patients undergoing cardioverter defibrillator implantations [47]. These variances in scores may be related to differences in age and comorbidities among the groups or could be a reflection of the higher rates of mood disturbances found in chronic low back pain patients.

Overall perceived health was unchanged after surgery when compare to preoperative level. This finding may be related to comorbidities. No other studies were found that reported overall perceived health in the back surgery population. Including a comorbidity measure in future studies may help clarify the role of comorbidities in perceived health.

**Figure 2:**
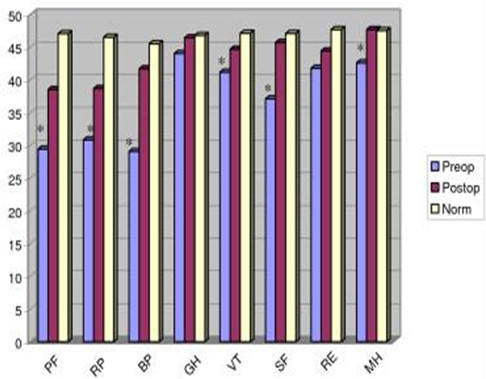
**SF12v2 preoperative, postoperative and norm comparisons**. * Indicates p < .05 between preoperative and postoperative scores. Higher scores reflect higher functioning. PF = physical functioning, RP = role-physical, BP = bodily pain, GH = general health, VT = vitality, SF = social functioning, RE = role-emotional, MH = mental health.

Health-related quality of life as measured by the SF-12v2 was significantly improved after surgery in terms of the perceived physical component summary (PCS) but not the perceived mental health summary (MCS). The improvement in the physical component can be attributed to decreased level of pain and better functional status. However, despite reporting an improved health-related quality of life three months after surgery, perceived physical health in this sample was lower than published norms for patient with low back pain (Figure 2) [32]. These lower scores were attributed to lower levels of physical function, vitality and physical role functioning when compared to normative data. Continuing recovery three months postoperatively may be one explanation for lower function and vitality as compared to the normative sample of non-surgical back pain subjects. There did not appear to be a difference in perceived mental health between this sample and the normative group.

As postoperative fatigue and lack of vitality were identified as lower than published norms for both the POMS-Brief Form and the SF-12v2, further research exploring levels of fatigue and lack of vitality during recovery from lumbar spinal surgery may be warranted. In addition it may be helpful for clinicians to be aware of when patients are expected to reach maximal improvement in levels of vitality and energy following lumbar spinal surgery so that rehabilitation and return to work activities can be appropriately timed. Although no studies were found related to level of fatigue in postoperative lumbar spinal surgery patients, several studies considered fatigue in patients with low back pain. For example, Fishbain et al [48] found that patients with chronic low back pain were significantly more fatigued than a non-patient control group. Furthermore, Fishbain et al [48] found that higher levels of fatigue were predicted by pain, female gender, depression and number of psychiatric comorbidities in chronic low back pain patients. In another study of 457 patients with low back pain compared to a normative sample, Hagen et al [15] found that low back pain sufferers reported more sleep disturbances related to pain, depression and anxiety. Causes and comorbidities of fatigue in low back pain patients may be different than those found in postoperative lumbar spinal surgery patients. However, measurement of sleep disturbances, depression, anxiety, as well as a comprehensive assessment of pain may be helpful in determining what factors are associated with fatigue in lumbar spinal surgery patients. In addition, longitudinal measurements of fatigue after lumbar spinal surgery may assist in determining the recovery trajectory in these patients.

There are several limitations of our study. The most significant limitation was the small sample size. A larger sample size may have demonstrated significant improvement in the mental health component of the SF-12v2. Another significant limitation of this study was the inclusion of various types of lumbar surgical procedures for herniated disks, degenerative changes and spinal stenosis. A wide age range as well as other variables may have influenced findings. For example, severity of disease and the presence of comorbidities may have impacted HRQOL ratings. A more homogeneous group would have allowed for better interpretation of findings. In addition, future studies should consider different surgical techniques such as the increasingly common minimally invasive approach. Finally, a control group of non-surgical low back pain patients may be helpful in determining whether or not changes in measured variables could be attributed to lumbar spinal surgery.

## Conclusion

Despite its limitations, the findings of this study contribute to the body of healthcare in several ways. It is the first known study that considered discrete aspects of HRQOL within an overall QOL framework in the lumbar spinal surgery population. Given the complexity of HRQOL measurement, it is important that a QOL framework be utilized in order to identify important variables to be measured. In addition, this study identified fatigue as a possible issue during recovery from lumbar spinal surgery which may have implications for rehabilitation. Lastly, the consideration of HRQOL outcomes following lumbar spinal surgery from the patient's perspective will assist clinicians in better meeting the needs and expectations of patients during the recovery period.

## Abbreviations

ANOVA-Analysis of variance; BMI-Body Mass Index; HRQOL-Health-Related Quality of Life; MANOVA-Multivariate analysis; MCS-Mental Component Summary; MOS-Medical Outcomes Study Social Support Survey; NPRS – Numeric Pain Rating Scale; ODI-Oswestry Disability Index; PCS-Physical Component Summary; POMS – Profile of Mood States; QOL-Quality of Life

## Competing interests

The author(s) declare that they have no competing interests.

## Authors' contributions

KLS: Participated in the conception and design of study, coordinated the study and drafted manuscript.

SMP: Participated in the conception and design of study, edited manuscript

IA: Participated in the conception and design of study, edited manuscript

FBB: Participated in the conception and design of study, edited manuscript
